# Editorial: LncRNAs in tumor microenvironment: its role in immunoregulation and inflammation

**DOI:** 10.3389/fcell.2025.1560928

**Published:** 2025-02-06

**Authors:** Dipanjan Ghosh

**Affiliations:** Department of Natural Products, National Institute of Pharmaceutical Education and Research, Kolkata, Kolkata, India

**Keywords:** lncRNA, cancer, tumor microenvironment, cancer therapy, inflammation

LncRNAs, a subset of non-coding RNA molecules characterised by its length of more than 200 nucleotides, have been shown to modulate biological processes through their complex multi-molecular interactome. However, such interactions also imply that lncRNAs act as regulators of multiple pathophysiological signalling pathways that contribute to cardiovascular disease, renal disease, inflammatory diseases, diabetes, and cancer. Particularly in cancer, lncRNAs not only control important cellular events such as proliferation, epithelial-mesenchymal transition (EMT), angiogenesis, immune escape, chemosensitivity and metastasis but also shape the tumor microenvironment (TME) through intracellular and extracellular association with several immune components such as tumor-associated macrophages (TAMs) and tumor-infiltrating lymphocytes (TILs) from the initial stages of the disease (Roy et al.). For instance, Pushkar Malakar’s study suggests a correlation of lncRNA PURPL (p53 upregulated regulator of p53 levels) with chromosomal instability (CIN)/aneuploidy as treatment with cell division blockers such as reversine, cytochalasin-B, aurora kinase inhibitor ZM447439 upregulated PURPL expression with a concomitant increase in CIN markers like p21 and MDM2 and knockout of PURPL promote micronuclei formation, deformed nuclear shape, and increased sensitivity towards CIN/aneuploidy inducers in different cancer cell types like 293T and MDA-MB-231. Furthermore, p53 upregulation and reversible knockdown affect PURPL levels in PURPL levels in RPE-1 cells when compared to p53 mutant DLD1 cells, hinting at an intertwinement of p53 and PURPL expression in CIN phenotype (Malakar). Another lncRNA, ST8SIA6-AS1, emerged as a novel target in hepatocellular carcinoma (HCC). ST8SIA6-AS1 increase cell proliferation, migration and invasiveness in liver cancer cells, inhibit apoptosis, promote tumor growth and metastasis and interact with viral factors in HBV-infected liver cancer cells. Moreover, the ability of ST8SIA6-AS1 to act as ceRNA (competing endogenous RNA), competitively interacting with miRNAs, builds on its growing popularity as a key player in liver cancer. The non-coding RNA molecule competitively bind to miRNAs like miR-142-3p, miR-651-5p, miR-338-3p, miR-129-5p, miR-5195-3p and miR-4656, aggravating HCC by increasing expression of cancer-related markers like HMGA1, TM4SF4, NONO, MEPCE, DCAF4L2, HOXB6, and HDAC11 (Qiu et al.). These findings make lncRNAs a tool in the prognosis of cancer as well as an attractive target for cancer therapy. Several lncRNAs have been identified as diagnostic markers for early detection of cancer and multiple therapeutic compounds, both natural and synthetic, demonstrate significant anticancer effects via modulation of lncRNA expression (Roy et al.). The rise of machine learning and computational biology further paved the way for the easy identification of lncRNA candidates acting as biomarkers and therapeutic targets. The study by Cui, Zhang and Lu et al. uses machine learning to identify novel exosome-derived lncRNA signatures in ovarian cancer (OC). Out of 420 OC patient samples in the TCGA used in the study, the low-risk group have shown increased levels of immune cell population and is associated with higher infiltration of activated CD4 T cell, activated CD8 T cell, effector memory CD8 T cell, immature B cell, gamma delta T cell, natural killer cell, natural killer T cell, plasmacytoid dendritic cell, Type 2 T helper cell, in addition to immature dendritic cell, insinuating the role of immune activation on tumor suppression. 11 lncRNAs are identified *in silico* to have increased expression in OC, i.e., COLCE-AS, TYMSOS, LEMD1-AS1, LINC00892, LINC00702, LINC02362, AC010834.3, WAC-AS1, AL391832.3, AC073389.2 and AC009244.1, whereas six lncRNAs, AC134312.1, AC010834.3, LEMD1.AS1, PCOLCE.AS1, LINC00892, and AL138820.1 demonstrate higher expression in the exosomes of OC cell lines *in-vitro* (Cui et al.). Another study employing computational biology found novel risk signatures based on coagulation-related lncRNAs (CRLs) in the immune cells of colorectal cancer (CRC) patients. Out of the 10 CRLs analysed, four lncRNAs, i.e., EWSAT1, LINC00645, LINC00901 and LINC02962, have been implicated with the promotion of malignancy, proliferation, EMT, growth and invasion of CRC. To add to the problem, genes such as CD96, IDO1, IL10, KDR, LAG3, TGFB1, and TIGIT show increased expression in the high-risk group, supporting the hypothesis of immunosuppressive TME driving CRC progression (Zhang et al.). LncRNAs shaping the immune microenvironment in cancer is a relatively new niche of research, and the advent of AI, machine learning, and computational biology, along with the improvement of existing *in-vitro* experimental techniques, may help in the deciphering of therapeutic applications of the lncRNA-TME interactome.

